# Establishment of Food Allergy Model in Dextran Sulfate Sodium Induced Colitis Mice

**DOI:** 10.3390/foods12051007

**Published:** 2023-02-27

**Authors:** Bihua Chen, Yuhong Wu, Huan Wu, Xuanyi Meng, Hongbing Chen

**Affiliations:** 1 State Key Laboratory of Food Science and Technology, Nanchang University, Nanchang 330047, China; 2 College of Food Science and Technology, Nanchang University, Nanchang 330031, China; 3 Jiangxi Province Key Laboratory of Food Allergy, Nanchang University, Nanchang 330047, China; 4 Jiangxi-OAI Joint Research Institute, Nanchang University, Nanchang 330047, China

**Keywords:** IBD, food allergy, mouse model, DSS colitis

## Abstract

Food allergy (FA) has become a global food safety issue. Evidence suggests that inflammatory bowel disease (IBD) can increase the incidence of FA, but it is mostly based on epidemiological studies. An animal model is pivotal for unraveling the mechanisms involved. However, dextran sulfate sodium (DSS)-induced IBD models may cause substantial animal losses. To better investigate the effect of IBD on FA, this study aimed to establish a murine model to fit both IBD and FA symptoms. Firstly, we compared three DSS-induced colitis models by monitoring survival rate, disease activity index, colon length, and spleen index, and then eliminated the colitis model with a 7-day administration of 4% due to high mortality. Moreover, we evaluated the modeling effects on FA and intestinal histopathology of the two models selected and found the modeling effects were similar in both the colitis model with a 7-day administration of 3% DSS and the colitis model with long-term administration of DSS. However, for animal survival reasons, we recommend the colitis model with long-term administration of DSS.

## 1. Introduction

Food allergy (FA), an adverse reaction caused by a specific immune response that repeatedly occurs after exposure to a certain food, has become a global food safety issue [1]. Australia has the highest prevalence of IgE-mediated FA, while other developed areas, such as Europe and the United States, have an estimated prevalence of 1–5% [2]. As our understanding of FA has grown, it has been discovered that gastrointestinal disorders can increase the occurrence of FA by altering intestinal permeability as well as the immune system’s mucosal barrier [3,4,5].

Inflammatory bowel disease (IBD) is an important gastrointestinal disease with an increasing prevalence that is categorized into Crohn’s disease (CD) and ulcerative colitis (UC). Several researchers have found many similarities between IBD and FA in terms of epidemiology, pathogenesis, and clinical course. In addition, IBD is often accompanied by allergic diseases [6,7], and the prevalence of FA is significantly higher in IBD patients than in healthy populations [8,9,10,11,12]. For example, Grzybowska et al. [8] investigated 95 IBD patients (2–18 years old) and found that 32.5% of UC patients and 21% of CD patients showed IgE-dependent FA. Moreover, Imanzadeh et al. [9] found that 60.7% of children with IBD (*n* = 28) suffered from FA, 35.7% of whom had multiple FAs. Additionally, Johnson et al. [12] tested the specific IgE levels of food allergens and inhalant allergens in the plasma of IBD patients (*n* = 29 UC, *n* = 37 CD) and healthy controls (*n* = 100) and found that UC patients were more sensitive to both food allergens and inhalant allergens, whereas CD patients were more sensitive to inhalant allergens only. In general, IBD is a high-risk factor for the development of FA, but studies on the relationship between IBD and FA have only focused on epidemiological data, and no comprehensive theoretical or clinical reports on the mechanisms underlying the association exist. Thus, a well-designed animal model, which can provide a sound basis to explore the effect of IBD on FA in humans, is very important.

To date, a great number of protocols to induce FA in mice have been published [13]. In experiments with mouse models of FA, several considerations are important, such as the genetic predisposition to allergy or tolerance of the mouse strain used, allergen dose, route of exposure (oral, intranasal, intraperitoneal, intradermal), damage to the epithelial barrier, use of adjuvants, food matrix effects, and composition of the microbiota [14]. Feeding food antigens to mice usually leads to oral tolerance, and classical isomorphic FA models require the co-administration of antigens with Th2-skewing agents (e.g., aluminum hydroxide, cholera toxin, or staphylococcal enterotoxin B) to counteract the normal tolerance response [15]. These models help us understand the transcriptional, cellular, and humoral characteristics of type 2 immune responses and the effector phase of FA; however, they do not contribute accurate information about the natural sensitization mechanisms that occur in humans [15]. Mouse models of FA should ideally be as similar as possible to the human disease. Epidemiological studies have established a strong correlation between atopic dermatitis (AD) and the development of FA [16]. There is evidence that transdermal exposure to food proteins in humans, especially during infancy or childhood, may lead to sensitization [17,18,19]. Indeed, dermal exposure in mice more effectively triggers food sensitization than the intragastric, intranasal, or sublingual routes [14]. As a result, we chose Noti et al. [20]’s mouse model of FA in which mice were sensitized to food antigens on a developing AD-like skin lesion induced by topical treatment with the vitamin D analog MC903.

A variety of IBD animal models have been established, including the chemically-induced colitis model, the adoptive transfer model, the spontaneous colitis model, and the genetically engineered/transgenic animal model. Among these models, the dextran sulfate sodium (DSS)-induced colitis model is the most widely used because of its widespread availability and low cost [21]. DSS-induced severe colitis is characterized by weight loss, hemorrhagic diarrhea, ulcer formation, epithelial cell loss, and neutrophil infiltration, similar to human UC episodes [22]. The efficacy of DSS-induced colitis depends on several factors, including the concentration of DSS (typically 1–5%), the duration and frequency of administration, the molecular weight of the DSS, the animal strain, and the microbial environment (germ-free versus specific pathogen-free) [23]. According to these different factors, DSS colitis may develop acute colitis, chronic colitis, or even colitis-induced dysplastic lesions in conventional animals [24]. However, these may cause severe weight reductions or animal losses. In this study, female BALB/c mice were used as this is the most frequently used strain in IBD and FA models and because females are more appropriate for long-term experiments as they are less aggressive than male animals [25]. In addition, a study reported a higher IgE response in female mice compared to male mice [26].

In this study, we induced colitis in mouse models with 7-day administration of DSS using the two most commonly used DSS concentrations (3 and 4% DSS) and a colitis model with long-term administration of DSS designed by Hoffmann et al. [24]. Then we established a FA model in DSS colitis mice. Finally, we choose one that reflects the clinical symptoms of IBD and FA without extensive weight loss or death. Therefore, this study aimed to establish a murine model to fit both IBD and FA symptoms without extensive animal loss.

## 2. Animals, Materials and Methods

### 2.1. Animals

A total of 32 7–8-week-old SPF female BALB/cAnNCrl mice (Charles River, Zhejiang, China, permission number “SCXK (Zhe) 2019-0001”) were kept in an animal room that was under a 12-h light/dark cycle at a temperature of 23 ± 1 °C and a humidity of 54 ± 2% and used for experimentation after 7 days of acclimatization. The mice were housed in plastic cages (310 × 230 × 160 mm) with SPF wood bedding material (Beijing Keao Xieli Feed Co., Ltd., Beijing, China), and fed on SPF standard pellet chow (Beijing Keao Xieli Feed Co., Ltd., Beijing, China). Animal experiments were performed according to protocols approved by the Institutional Review Board of the Second Affiliated Hospital of Nanchang University under their guidelines (permission number “20210311-028”).

### 2.2. DSS-Induced Colitis

**Figure 1:**
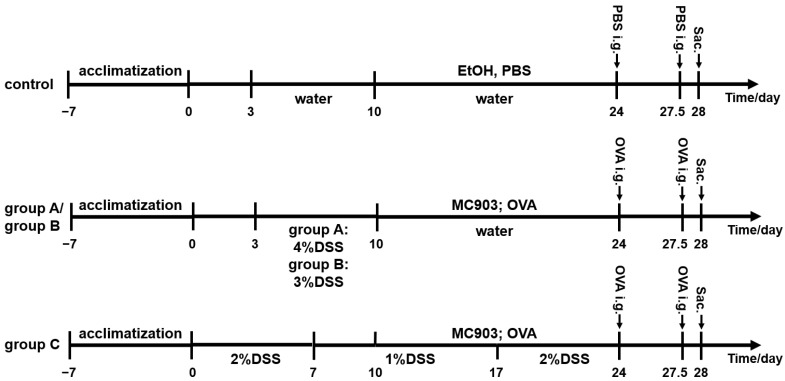
The experimental protocol of 4 groups. group A: a group with a 7-day administration of 4% DSS; group B: a group with a 7-day administration of 3% DSS; group C: a group with a long-term administration of DSS. DSS: dextran sulfate sodium; OVA: Ovalbumin; i.e.: intragastrical administration; Sac.: sacrifice.

The experimental protocol is shown in Figure 1. The 32 mice were randomly divided into four groups, including a control group (*n* = 8), a group with a 7-day administration of 4% DSS (group A, *n* = 8), a group with a 7-day administration of 3% DSS (group B, *n* = 8), and a group with a long-term administration of DSS (group C, *n* = 8). Mice in the control group were given autoclaved water throughout the experiment. Mice in group A were given 4% DSS (molecular weight 36,000–50,000 Da, MP Biomedicals, Santa Ana, CA, USA) in autoclaved drinking water (w/v) for 7 days, followed by DSS-free drinking water. Mice in group B were given 3% DSS in autoclaved drinking water for 7 days, followed by DSS-free drinking water. Mice in group C were induced by the administration of 2% DSS for 7 days, followed by 1% DSS for 10 days and 2% DSS for another 7 days. Water intakes were determined as described by Anderson et al. [27]. Animals were inspected daily for survival rate, body weight, stool consistency, and colonic hemorrhage. In this experiment, the survival rate was defined as the absence of mouse mortality or the loss of mice to 20% of their pre-experimental body weight during the experiment. Mice were euthanized when they had lost 20% of their pre-experimental body weight. The disease activity index (DAI) score was calculated as the sum of the weight loss, stool consistency, and blood in stool scores (Table 1).

### 2.3. Experimental FA

The experimental protocol is shown in Figure 1. To induce experimental FA, both ears of mice were treated with 2 nmol of MC903 (LEO Pharmaceutical Products Ltd., Ballerup, Denmark) in 20 µL of 100% EtOH for 14 consecutive days. 100 mg of ovalbumin (OVA, Sigma, St. Louis, MO, USA) in phosphate buffered saline (PBS; Solarbio, Beijing, China) was applied on dry ears after MC903 treatment. The same volume of EtOH and PBS was applied to mice in the control group. All mice were fasted for 12 h before the oral challenge with 250 µL of 200 mg/mL OVA in PBS on days 24 and 27.5 and euthanized 12 h after the second oral challenge. Mice in the control group were treated with the same volume of PBS. The body temperatures of mice were measured with a rectally inserted thermal probe before the challenge and 20, 35, and 50 min post-challenge. Starting 20 min after the oral challenge, mice were individually observed for 30 min and assigned a clinical allergy score described by Hussain et al. [28]. Briefly, the allergy score (Table 2) was calculated as the total value of the scratching, behavior, physical appearance, and stool consistency. When conducting food allergy scores, observers were blinded to the treatment group.

### 2.4. Measurement of Colon Length and Spleen Index

On the day of termination, euthanasia was performed under deep anesthesia using carbon dioxide after terminal bleeding was stopped using orbital blood collection. The spleen was collected and weighed, and the spleen index was calculated based on the following formula: spleen weight (mg) × 10/body weight (g). The colon was dissected, and the length was measured from the cecum to the anus. After dissection, the small intestine (jejunum) tissue and colonic tissue were collected.

### 2.5. Enzyme-Linked Immunosorbent Assay (ELISA)

Blood was collected from mice and stored at 4 °C for 12 h before serum was collected and stored at a −80 °C for use. Measurements of histamine levels in serum were performed by a commercially available ELISA kit (Elabscience, Wuhan, China) using the competitive-ELISA principle. The levels of allergen-specific IgE, IgG, IgG1, and IgG2a in serum were determined using an indirect ELISA, as previously described [29]. 100 µL of 40 μg/mL OVA in 50 mM sodium carbonate buffer (pH 9.6) was added to a 96-well microtiter plate and incubated at 4 °C overnight. Serum was diluted with PBS (1:100 for IgE, 1:2000 for IgG and IgG1, and 1:50 for IgG2a), and 100 μL of the diluted serum was added to each well.

### 2.6. Histological Analysis

For histological analysis, colon (at about 1 cm from the anus) and small intestine (jejunum) tissues were fixed in 4% paraformaldehyde for 24–48 h and then embedded in paraffin, and sections were separately stained with hematoxylin & eosin (H&E) and toluidine blue (TB). The length of the intestine samples was around 1 cm, and two sections per sample were analyzed in each staining. Sections were observed using a light microscope (Nikon, Tokyo, Japan). Colon histological scoring was performed using the method described by Kovacs-Nolan et al. [30] Briefly, the colon histological scores (Supplementary Table S1) were calculated as the total value of the degree, extent, and depth of inflammation, as well as the amount of damage to the glands and epithelium. Small intestine histological scores were measured according to the method described by Kim et al. [31] Briefly, the small intestine histological score (Supplementary Table S2) was determined by the degree and extent of damage to structural components (such as the epithelial layer, lamina propria, and villi).

### 2.7. Immunohistochemistry

Myeloperoxidase (MPO) is a heme-peroxidase that mostly exists in neutrophils, making up roughly 5% of the total dry cell weight of neutrophils, while other cell types such as monocytes and certain macrophage subpopulations contain a much lesser extent of MPO [32]. Therefore, in this study, we used MPO to target neutrophils. MPO expression levels in the small intestine and colon were assessed by immunohistochemistry. Paraffin sections were dewaxed by successive immersions in xylene and ethanol. The antigen was then repaired at high temperature and pressure using 1× citric acid (pH 6.0) as the repair solution. The sections were incubated with 10% goat serum for 30 min at 37 °C and with MPO antibody (Abcam, Cambridge, England) overnight at 4 °C. The secondary antibody was prepared in PBS-Tween 20 at a dilution ratio of 1:100 and incubated at 37 °C for 1 h. Finally, DAPI working solution was added dropwise to the tissue and incubated at room temperature for 5 min, then washed with PBS three times for 5 min. The liquid was shaken dry, and an anti-fluorescent blocker was added dropwise to the tissue and covered with a coverslip.

### 2.8. Statistical Analysis

All data were analyzed using GraphPad Prism 9.0.0 software (GraphPad Software, La Jolla, CA, USA). The results are presented as mean ± SEM. Statistical significance was determined using the Student’s t test or a 2-way ANOVA with Tukey multiple comparison tests. Results were defined as significant at *p* values less than 0.05. The number of experiments performed is indicated at the end of each figure legend.

## 3. Results

**Figure 2:**
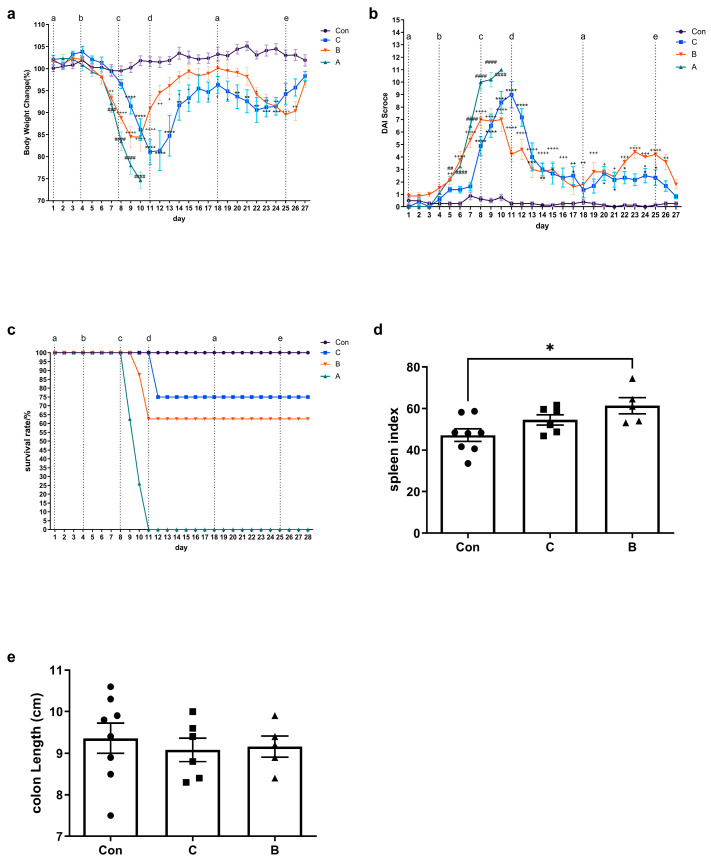
Long-term administration of DSS is more suitable. (**a**) survival rate, (**b**) body weight change, (**c**) DAI score of mice, (**d**) spleen index, and (**e**) colon length of mice. The percentage of body weight change was calculated relative to the body weight at day 0. The value was evaluated daily and is shown as the mean ± SEM for 5–8 animals per group. The survival rate was defined as the absence of mouse mortality or the loss of mice to 20% of their pre-experimental body weight during the experiment. The spleen index was calculated according to the following formula: spleen weight (mg) × 10/body weight (g). For (**a**–**c**): group C vs. the control group, * *p* < 0.05, ** *p* < 0.01, *** *p* < 0.001, **** *p* < 0.0001; group B vs. the control group, + *p* < 0.05, ++ *p* < 0.01; +++ *p* < 0.001; ++++ *p* < 0.0001; group A vs. the control group, ## *p* < 0.01, ### *p* < 0.001, #### *p* < 0.0001. a: group C started to drink autoclaved water with 2% DSS; b: group B/A initiated drinking autoclaved water at 3% DSS or 4% DSS; c: group C commenced drinking autoclaved water with 4% DSS; d: returned to drinking autoclaved water; e: group C began drinking autoclaved water. For (**d**,**e**): ● represents the individual in group con, ■ represents the individual in group C, ▲ represents the individual in group B, * *p* < 0.05.

The colitis model with long-term administration of DSS is a more suitable model. For the IBD mouse model, we compared a group with a 7-day administration of 4% DSS (group A), a group with a 7-day administration of 3% DSS (group B), and a group with a long-term administration of DSS (group C). The survival rates (Figure 2a) of the groups were recorded daily, and we found that the losses in group A were severe, with 100% mortality on day 11. Thus, we eliminated the colitis model with a 7-day administration of 4% DSS. Survival rates in groups C and B were 75 and 62.5%, respectively, which were within the acceptable range. Clinical scores (Figure 2b,c) based on weight loss, stool consistency, and colonic hemorrhage were assessed daily. IBD symptoms were similar in groups B and C. Body weight dropped significantly on days 7 for group B and 9 for group C. Group B had the most severe symptoms on experimental day 10 (day 7 of 3% DSS administration), but they gradually subsided after resumption of administration of autoclaved water. Group C had the most severe symptoms on experimental day 11 (after 7 days of 2% DSS administration and 4 days of 1% DSS administration), but they subsequently diminished. Colon length (Figure 2d) decreased slightly in groups C and B, but the difference was not significant compared with the control group. The increased spleen weight was generally correlated with the degree of inflammation [33]. Therefore, the spleen index (Figure 2e) was measured. Compared to the control group, the spleen index was higher in group B (*p* < 0.05). The relative increase was also higher in group C than in the control group, but the difference was not significant.

**Figure 3:**
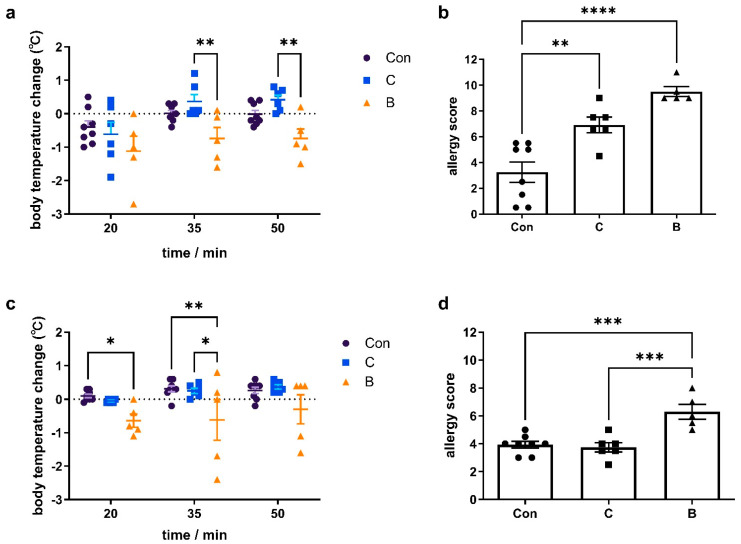
Groups with a 7-day administration of 3% DSS (group B) showed more severe clinical symptoms of FA compared to groups with a long-term administration of DSS (group C). (**a**) body temperature change and (**b**) clinical allergy score after the first oral challenge; (**c**) body temperature change and (**d**) clinical allergy score after the second oral challenge. The change in body temperature was calculated based on the body temperature before the oral challenge. The allergy score was calculated as the sum of the values of the scratching, behavior, physical appearance, and stool consistency. The value was shown as the mean ± SEM for 5–8 animals per group. ● represents the individual in group con, ■ represents the individual in group C, ▲ represents the individual in group B, * *p* < 0.05, ** *p* < 0.01; *** *p* < 0.001; **** *p* < 0.0001.

**Figure 4:**
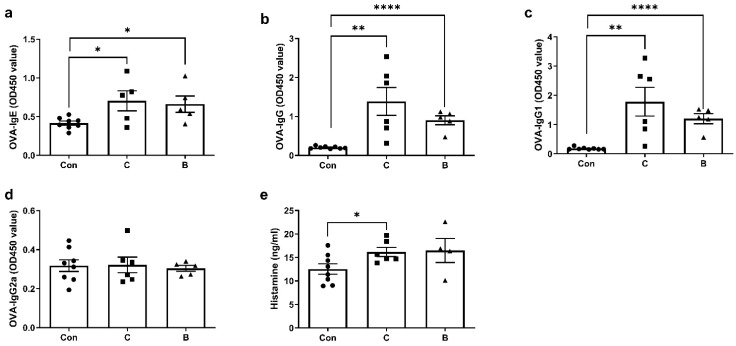
The serum levels of specific antibodies and histamine were similar in the group with long-term administration of DSS (group C) and the group with a 7-day administration of 3% DSS (group B). OVA-specific (**a**) IgE, (**b**) IgG, (**c**) IgG1 and (**d**) IgG2a levels, and (**e**) histamine concentration in mouse serum. The value was shown as the mean ± SEM for 5–8 animals per group. ● represents the individual in group con, ■ represents the individual in group C, ▲ represents the individual in group B, * *p* < 0.05 ** *p* < 0.01; **** *p* < 0.0001.

The colitis model with a 7-day administration of 3% DSS demonstrated the most stable relative performance for FA. On day 10, mice were sensitized to the food antigen OVA on AD-like skin lesions. Mice were then challenged intragastrically twice with OVA to induce FA. This resulted in FA phenotypes such as lower body temperatures (Figure 3a,c), higher clinical FA scores (Figure 3b,d), OVA-specific IgE, IgG, IgG1 and IgG2a responses (Figure 4a–d), and serum histamine levels (Figure 2e). After the second oral challenge, group B showed a significant decrease in body temperature after 20 and 35 min compared to the control group. However, group C did not exhibit a decreasing temperature trend after either the first or second challenge. Furthermore, compared with group C, group B showed a more significant increase in clinical symptom scores after both the first and second oral gavage challenge. Groups C and B both showed a significant increase in levels of serum OVA-specific IgE (group C: *p* < 0.05; group B: *p* < 0.05), IgG (group C: *p* < 0.01; group B: *p* < 0.0001), and IgG1 (group C: *p* < 0.01; group B: *p* < 0.0001), whereas no differences were found for OVA-specific IgG2a. Body temperature changes and the clinical allergy score both indicated that FA clinical symptoms were less severe in group C than in group B. However, serum histamine levels were significantly elevated in group C compared to the control group (*p* < 0.05), whereas no difference was observed in group A.

**Figure 5:**
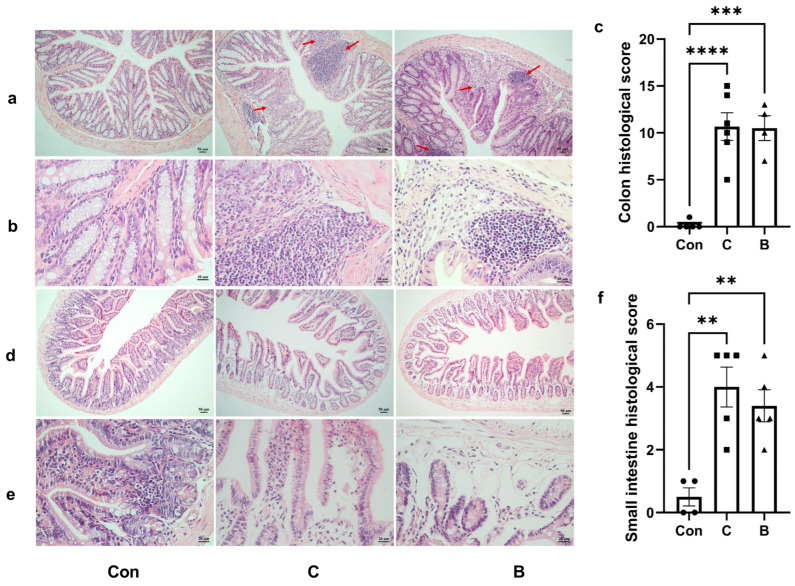
Group with long-term administration of DSS (group C) and group with 7-day administration of 3% DSS (group B) exhibit similar intestinal pathology. (**a**) HE colon staining (100× magnification); (**b**) HE colon staining (400× magnification); (**c**) histological score of colonic tissue; (**d**) HE small intestine staining (100× magnification); (**e**) HE small intestine staining (400× magnification); (**f**) histological score of small intestinal tissue. The value of the histological score was shown as the mean ± SEM for 5–8 animals per group. ● represents the individual in group con, ■ represents the individual in group C, ▲ represents the individual in group B, ** *p* < 0.01; *** *p* < 0.001; **** *p* < 0.0001.

**Figure 6:**
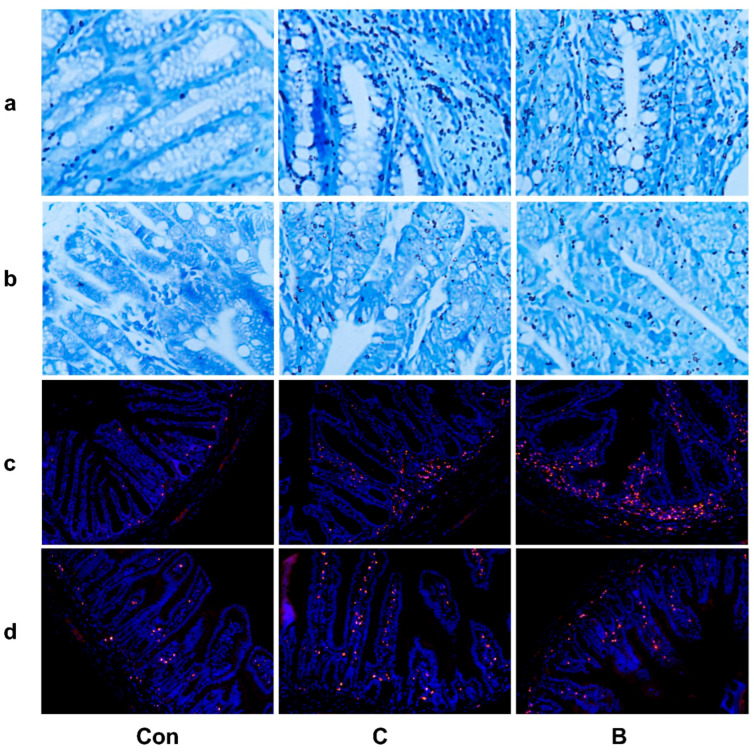
Groups with long-term administration of DSS (group C) and groups with 7-day administration of 3% DSS (group B) both exhibit mast cell and neutrophil infiltration. (**a**) TB colon staining (400× magnification); (**b**) TB small intestine staining (400× magnification); (**c**) representative images of MPO expression in colon tissue; (**d**) representative images of MPO expression in small intestine tissue. Mast cells in the small intestine and colon tissue are stained purple. MPO protein in small intestine and colon tissue is detected by immunohistochemical methods; positive cells are stained in red, and MPO-positive cells represent neutrophils.

The colitis model with 7-day administration of 3% DSS and the colitis model with long-term administration of DSS exhibit similar intestinal pathology. The severity of inflammation was assessed morphologically in the colon (Figure 5a,b) and small intestine (Figure 5d,e). Histopathological evaluation of colon tissue from both groups C and B indicated significant mucosal and submucosal damage, including multifocal gland dilatation, epithelial tufting and erosion, and infiltration of inflammatory cells, resulting in significantly increased histopathological scores (Figure 5c). The small intestines of mice in both groups C and B exhibited characteristics of injury, such as villous atrophy (Figure 5d–f). Mast cell activation was observed in both groups C and B when the colon (Figure 6a) and jejunum (Figure 6b) were stained with TB. Both groups had more mast cell activation in the jejunum than in the colon, but group C had higher levels of mast cell activation compared to group B. In addition, visualization of MPO-positive neutrophils in tissue sections of both the colon (Figure 6c) and small intestine (Figure 6d) illustrated neutrophil infiltration in mice in groups C and B. Overall, mice in groups C and B had damaged intestinal mucosa as well as mast cell and neutrophil infiltration, which is consistent with the pathological manifestations of our desired model.

## 4. Discussion

Evidence has been presented that IBD increases the incidence of FA, but it is based mostly on epidemiological studies. A convenient way to study the effects of IBD on FA in humans is to induce FA in IBD mice. Therefore, we selected three DSS-induced IBD models to explore the effects of IBD on FA. We first considered the survival rate of these three IBD models. Typically, a 7-day administration of 3 or 4% DSS is used to induce acute colitis, and we applied a procedure based on the chronic model established by Hoffmann et al. [25]. However, few survival rates have been recorded. In our study, the colitis model with 7-day administration of 4% DSS had a 100% mortality rate, the colitis model with 7-day administration of 3% DSS had a survival rate of 62.5%, and the survival rate of the colitis model with long-term administration of DSS was 75%. Therefore, although the colitis model with 7-day administration of 3% DSS and the colitis model with long-term administration of DSS showed similar weight loss, DAI, and colon length, the colitis model with long-term administration of DSS was more in line with our requirements. In addition, although body weight loss and DAI were significantly improved in both groups C and B at the end of the experiment, decreased colon length, increased splenic index, and significant colonic damage represented successful IBD modeling and were consistent with the disease relapse-remission process in clinical patients.

Because dermal exposure in mice triggers food sensitization more quickly and effectively than other routes, we selected the experimental FA model established by Noti et al. [20]. Consistent with the studies of Noti et al. [20] and Hussain et al. [28], mice showed increased allergy scores, serum IgE levels, and FA-related intestinal mast cell accumulation. Unlike the findings of Noti et al. [20], who observed no decrease in body temperature, mice in group B had significantly lower body temperatures after the second challenge in our experiment. However, it is not clear whether this was the result of colitis exacerbating the symptoms of FA. Specific IgE against a particular allergen is a key factor in immediate hypersensitivity [34]. Several allergen-specific IgG antibodies can lead to complement activation and production of C5a, which promotes the recruitment of several inflammatory immune cells (i.e., macrophages, eosinophils, basophils, and mast cells) as well as increased secretion of histamine, leukotrienes, platelet-activating factor, and various cytokines, which trigger many of the pathophysiological features of allergy [35]. Thus, significant increases in OVA-specific IgE, IgG, and IgG1 in both group C and group B indicate that the FA model was successful.

Both human and mouse colitis cause damage to the colonic mucosa, leading to ulceration, inflammatory cell infiltration, and reduced intestinal epithelial cells [21,36]. HE staining of colon tissue from mice in groups C and B revealed significant mucosal and submucosal damage, which was consistent with other studies [27,37]. Changes in the function and composition of the intestinal epithelial barrier have been observed in both human and mouse FA [3,30], as well as in the HE-stained small intestine tissue of mice in groups B and C. Abnormal activation of mast cells is common in both IBD and FA. It is well known that mast cells are the major effector cells in IgE-mediated FA. Moreover, mast cells can be activated by antigen-mediated crosslinking of immunoglobulin receptors, free light chains of immunoglobulins, stress, and adenosine triphosphate (ATP), which is followed by the release of bioactive mediators, of which the serine proteases mMCP-6 and Prss31 have been shown to be involved in the development of acute colitis [38]. In our study, increased mast cell numbers were observed in both the colon and small intestinal tissues of mice in groups B and C. Neutrophils are associated with the pathogenesis and development of intestinal mucosal inflammation in IBD. When intestinal immune tolerance collapses, large numbers of neutrophils accumulate in the inflamed mucosa, inducing damage to crypt structures and leading to the formation of cryptitis and crypt abscesses and increased intestinal mucosal inflammation [39]. In our experiment, both the colon and small intestine tissues of the experimental groups showed neutrophil infiltration, which is consistent with the clinical symptoms of IBD. In the case of FA, it has been documented that neutrophilia is significantly positively correlated with the development of gastrointestinal symptoms in patients with FA [40]. However, whether the intestinal neutrophil infiltration caused by IBD represents an aggravation of FA symptoms requires further investigation.

There are some limitations to this study. Firstly, only female mice were studied. Although male mice are less commonly used in FA research as they have a tendency to fight, sex as a biological variable can influence the immune response related to FA, including antibody production and T-cell function. Future research needs to be done on both sexes. In addition, this study was limited by only exploring the murine model to fit both IBD and FA symptoms without extensive animal loss. Future research should be undertaken to explore the effect of IBD on the development or susceptibility of FA. In conclusion, we present two models for establishing FA in mice with DSS-induced colitis. The colitis model with a 7-day administration of 3% DSS (group B) can be used for acute colitis, whereas the colitis model with long-term administration of DSS (group C) can be used for chronic colitis. Both models avoid high mortality rates but retain the most relevant IBD and FA symptoms. Because the modeling effects of IBD and FA were similar in both groups, we recommend that the colitis model with long-term administration of DSS be used for survival reasons. Moreover, the colitis model with long-term administration of DSS is more consistent with the disease relapse-remission process in clinical patients.

## Figures and Tables

**Table 1:** Disease Activity Index.

Parameter	Description	Score
Body weight loss (%)	None	0
	1–5%	1
	5–10%	2
	10–20%	3
	>20%	4
Stool consistency	Normal	0
	Soft but soft	1
	Loose	2
	Watery stool	3
	Severe diarrhea	4
Colonic hemorrhage	None	0
	Visible	2
	Large amount of blood in the stool	3
	Perianal bleeding	4

**Table 2:** Clinical Food Allergy Score [28].

Parameter	Description	Score
Scratching	No symptoms	0
	Mild scratching; rubbing of the nose, head, or feet (<5 episodes)	1
	Intermediate scratching; rubbing of the nose, head, or feet (>5 to <10 episodes)	2
	Severe scratching (>10 episodes)	3
Behavior	Normal	0
	Hyperactivity	1
	Aggressive behavior; pain is loud after prodding	2
Physical appearance	Normal activity	0
	Significantly reduced mobility; piloerection	2
	Immobility after prodding, tremors, and/or significant respiratory distress	3
Stool consistency	Normal	0
	Loose stool	2
	Diarrhea	4

## Data Availability

No new data were created or analyzed in this study. Data sharing is not applicable to this article.

## Supplementary Materials

The following supporting information can be downloaded at: https://www.mdpi.com/article/10.3390/foods12051007/s1. Table S1: The histopathology scoring system for colonic sections; Table S2: The histopathology scoring system for small intestinal sections.
