# Supplementary material for: Establishment of Food Allergy Model in Dextran Sulfate Sodium Induced Colitis Mice
Source: Foods. 2023 Feb 27;12(5):1007. doi: 10.3390/foods12051007 (PMC10001293; doi:10.3390/foods12051007)
Supplement: Supplementary file 1 [file foods-12-01007-s001.zip › foods-2103024-supplementary.pdf]

foods MDPI

Table S1. The histopathology scoring system for colonic sections [30].

Parameter	Description	Score
Inflammation	Normal	0
	Minimal infiltration of lamina propria, focal to multifocal	1
	Mild infiltration of lamina propria, multifocal, mild gland separation	2
	Moderate to mixed infiltration, multifocal with minimal edema	3
	Marked mixed infiltration into submucosa and lamina propria with extensive areas of gland separation	4
Epithelium	Normal	0
	Minimal: focal mucosal hyperplasia	1
	Mild: multifocal tufting of rafts of epithelial cells with increased numbers of goblet cells	2
	Moderate: extensive locally extensive to multifocal erosion or epithelial attenuation	3
	Marked: locally extensive mucosal ulceration	4
Glands	Normal	0
	Minimal: rare gland dilatation	1
	Mild: multifocal gland dilatation	2
	Moderate: multifocal gland dilatation with abscessation and occasional loss of glands	3
	Marked: locally extensive to subtotal loss of glands	4
Depth of lesion	None	0
	Mucosa	1
	Mucosa and submucosa	2
	Transmural	3
Extent of section affected	None	0
	Minimal: <10%	1
	Mild: 10–25%	2
	Moderate: 26–50%	3
	Marked: >50%	4

Table S2. The histopathology scoring system for small intestinal sections [31].

Description	Score
no damage	1
a low amount of damage, with distinct structural components (epithelial layer and lamina propria)	2
structural components can still be differentiated, but the epithelial layer is noticeably separating from the lamina propria	3
some disorganization of the villi and structural components are difficult to differentiate;	4
the structure of villi is chaotic and many villi are completely destroyed down to the basal layers of tissue	5
